# Supplementary figures and images for: AtFAHD1a: A New Player Influencing Seed Longevity and Dormancy in Arabidopsis?
Source: Int J Mol Sci. 2021 Mar 15;22(6):2997. doi: 10.3390/ijms22062997 (PMC8001395; doi:10.3390/ijms22062997)

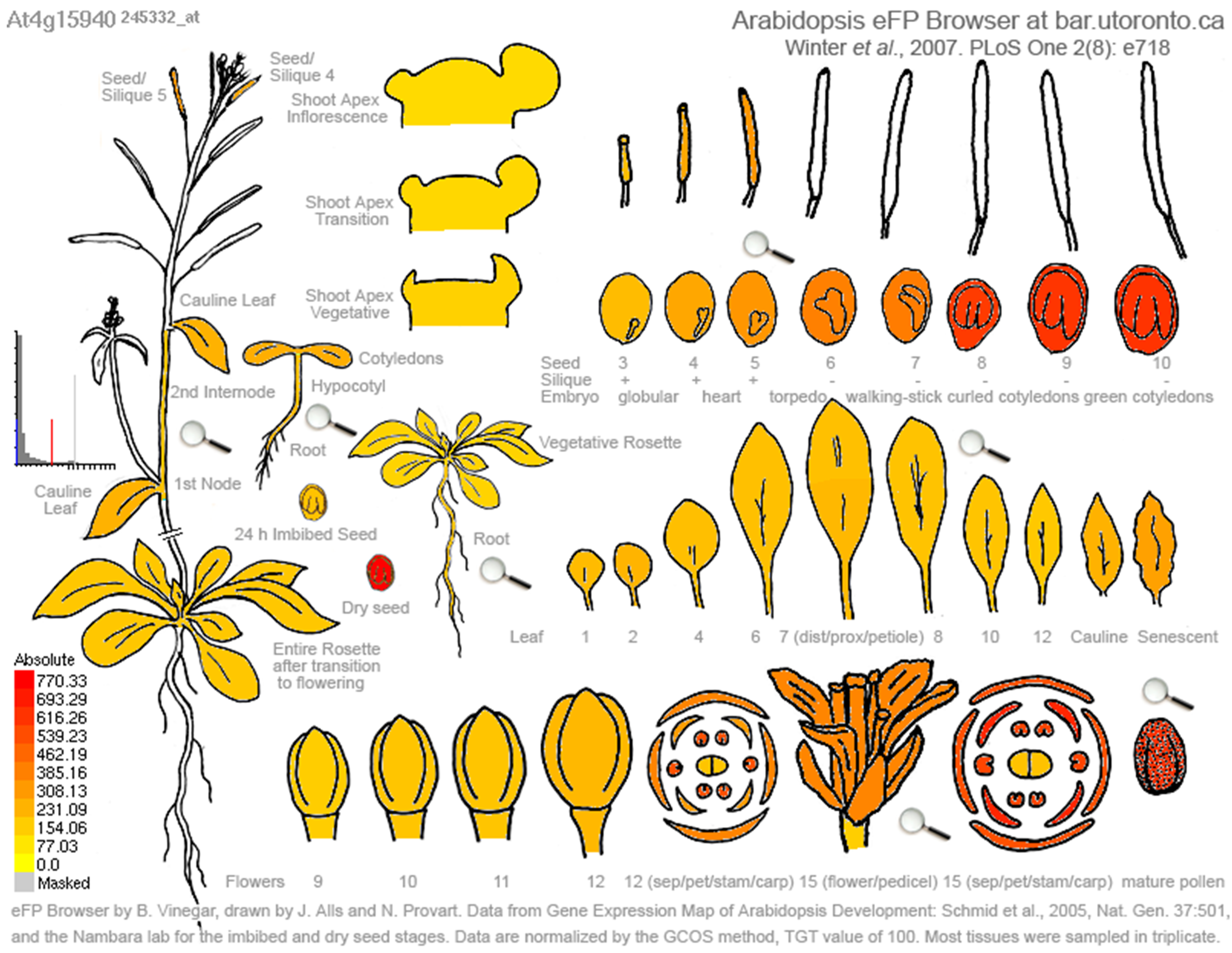

Supplement: Supplementary file 1 [file ijms-22-02997-s001.zip › Figure S1.tif]

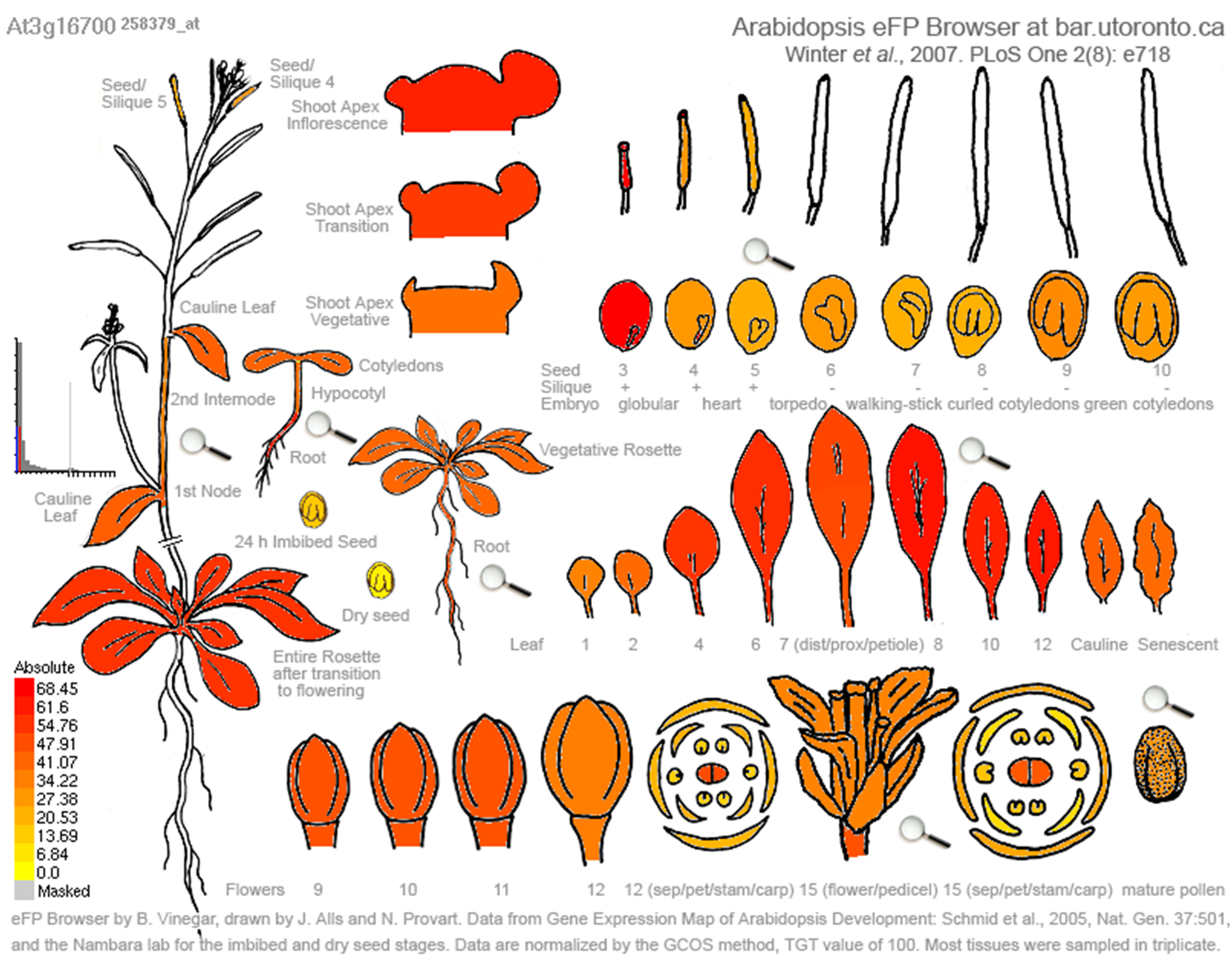

Supplement: Supplementary file 1 [file ijms-22-02997-s001.zip › Figure S2.tif]

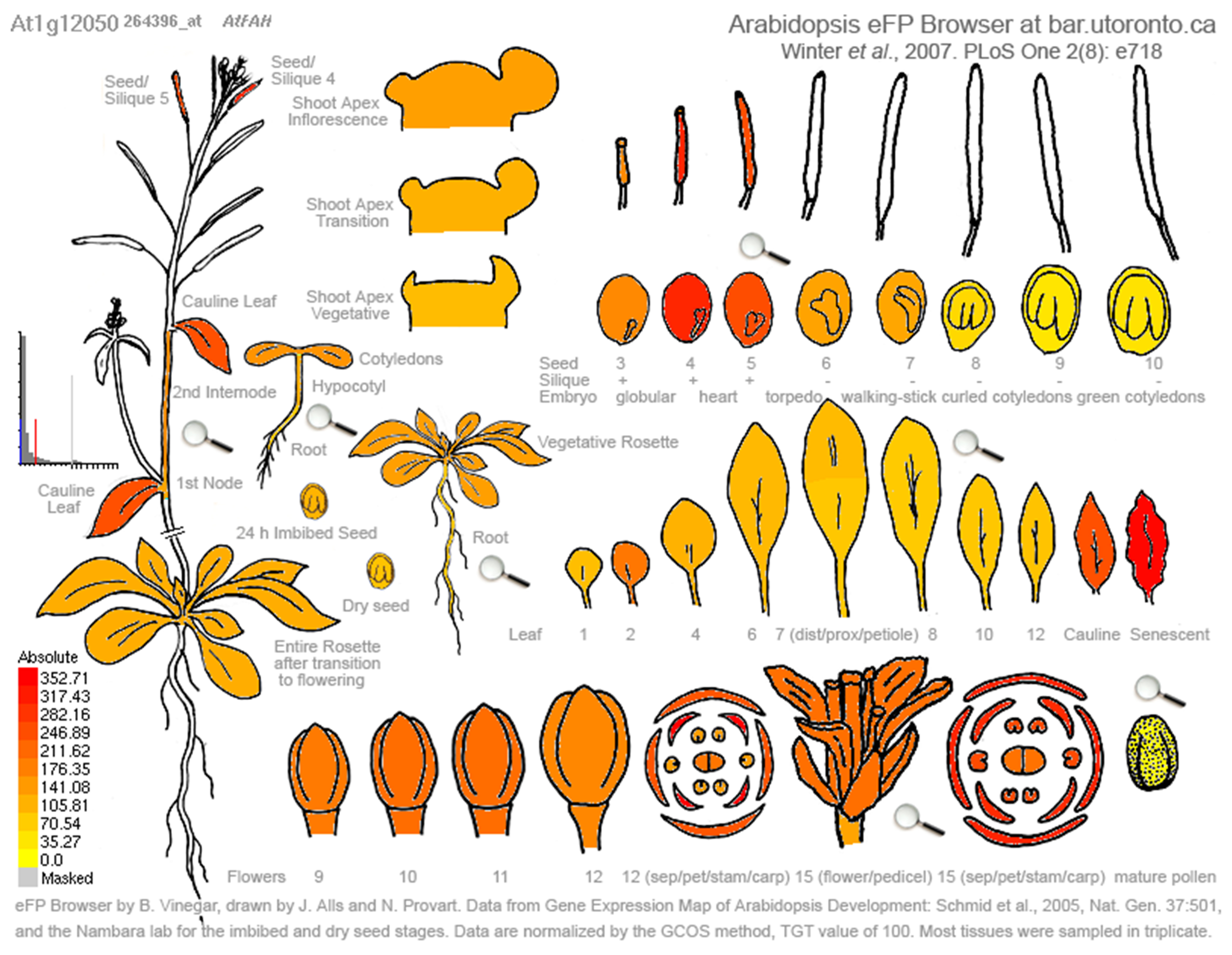

Supplement: Supplementary file 1 [file ijms-22-02997-s001.zip › Figure S3.tif]

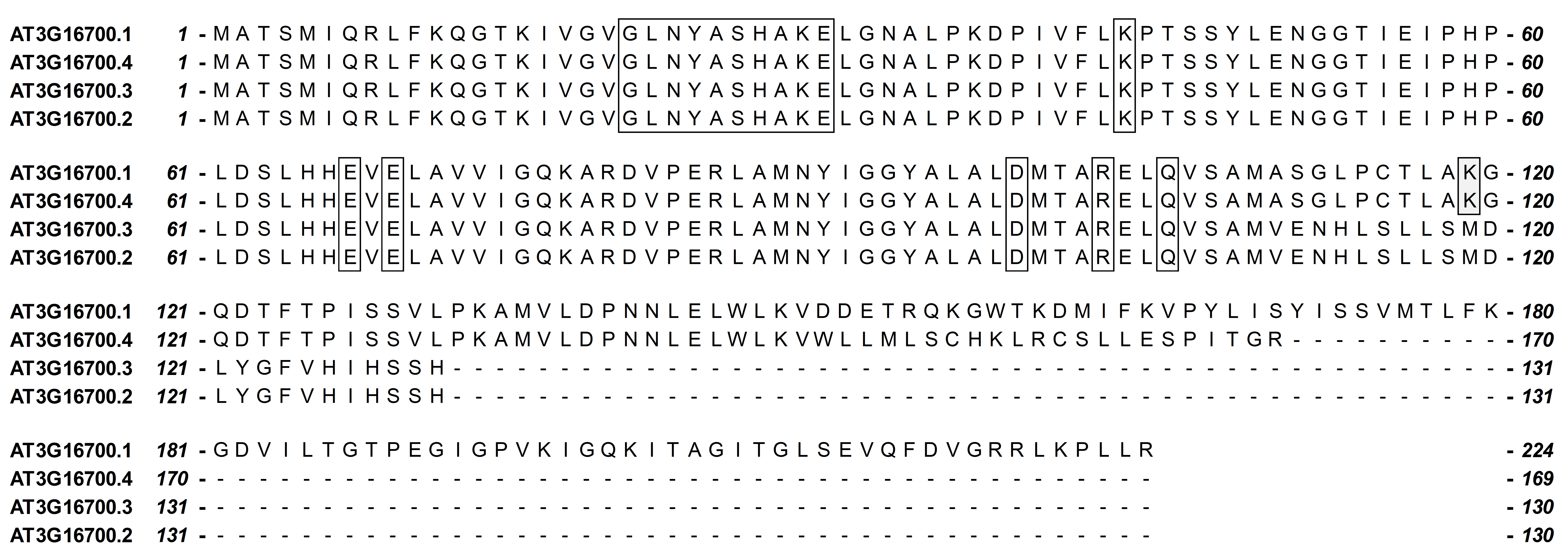

Supplement: Supplementary file 1 [file ijms-22-02997-s001.zip › Figure S4.tif]

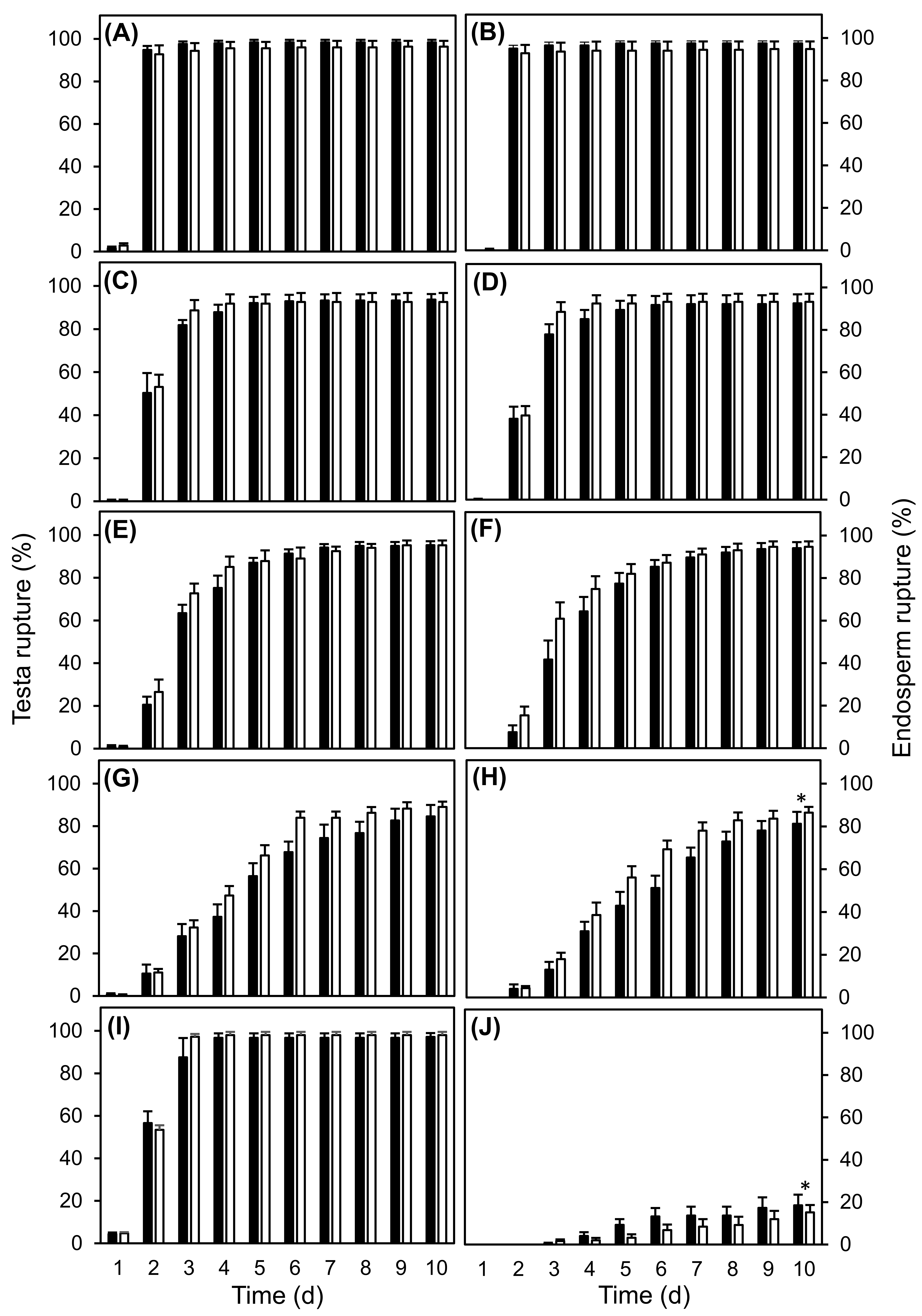

Supplement: Supplementary file 1 [file ijms-22-02997-s001.zip › Figure S5.tif]

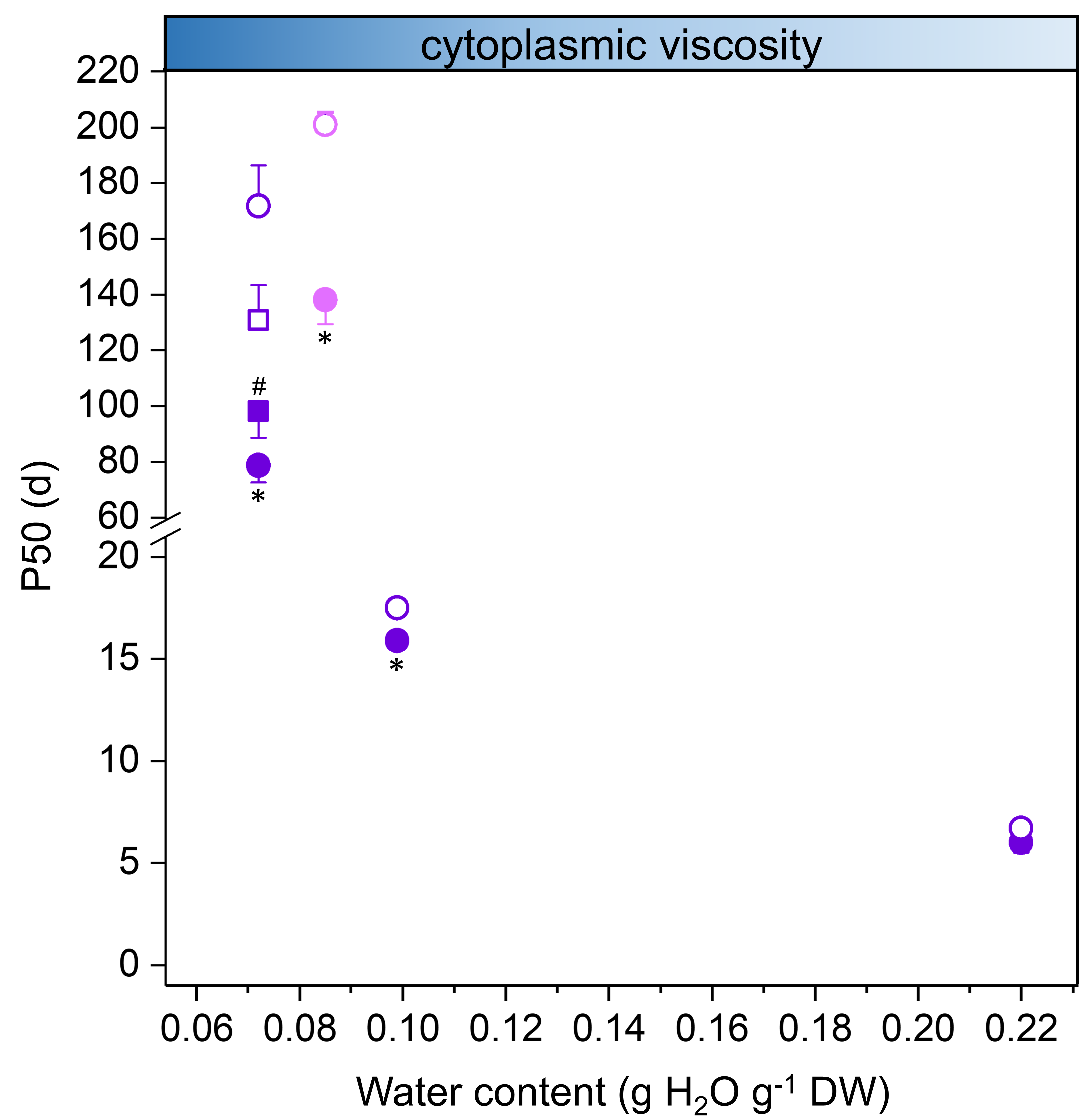

Supplement: Supplementary file 1 [file ijms-22-02997-s001.zip › Figure S6.tif]
